# Dynamic salience as a basis for behavioural meta-malleability

**DOI:** 10.1098/rsos.252449

**Published:** 2026-09-09

**Authors:** Rodrigo Sosa, Mario Treviño

**Affiliations:** ^1^ Escuela de Pedagogía y Psicología, Universidad Panamericana, Guadalajara, Jalisco, Mexico; ^2^ Instituto de Neurociencias, Universidad de Guadalajara, Guadalajara, Jalisco, Mexico

**Keywords:** associative learning, phenotypic plasticity, adaptive behaviour, infotaxis, epistemic actions, behavioural individuation

## Abstract

*Salience*, the degree to which cues capture attention, is not fixed but updated through experience. Learning which cues anticipate meaningful outcomes requires cue–outcome relationships to be perceptible to begin with, rendering salience a gate for behavioural change. Because salience is itself malleable, behaviour exhibits ‘meta-malleability’, whereby the modifier of behaviour (learning) can itself be modified. A cue's salience is determined partly by its physical properties and capacity to stand out, and partly by the subject's prior experience with it. Cues reliably followed by important outcomes capture more attention, as if they were physically intense, whereas poor predictors lose their attention-capturing potential even when physically intense. Concurrent cues compete for attention, and less salient cues are undermined by more salient counterparts. This competition occurs regardless of how each cue acquired its salience status. Under certain circumstances, organisms orient towards informative cues at the expense of biological needs; yet, they may sometimes orient towards cues with uncertain outcomes. Together, these findings demand an overarching framework. We synthesize behavioural, computational and neurobiological perspectives on salience dynamics. One pay-off of this framework is a principled account of why order matters across otherwise equivalent encounters with cues of varying salience, channelling behaviour down diverging trajectories.

## 1. Introduction

Behaviour in organisms is malleable. Agents change the way they interact with their surroundings whenever events do not align with what they expect based on past experiences. This process of updating our actions in response to discrepancies between anticipated and actual outcomes is known as learning through prediction error correction. However, learning about a cue that signals an outcome is constrained by the amount of attention agents allocate to both the target cue and other cues in their surroundings. Not all cues capture attention to the same degree; in general terms, the extent to which they do is known as *salience*. Salience emerges from a complex interplay between the intrinsic conspicuity of environmental cues [1] and the perceiver's prior experience.

Intense cues readily capture attention, out-competing equally informative but subtler cues. We define intensity as the magnitude of a stimulus, quantified in terms of physical energy. For visual cues, intensity may be related to the size, luminescence or saturation of particular locations in the retina. In the auditory system, it can be associated with the loudness of cues, while in proprioception, it corresponds to the force applied to the sensory fibres. In olfaction and taste, it is related to the concentration of particles in the sensed substance, and so on. Stimulus intensity generally correlates with neuronal firing rates; however, this relationship is rarely linear, as sensory systems often exhibit adaptation, saturation or other nonlinear mappings between stimulus energy and neural response [2]. In most cases, intensity corresponds directly to the detectability of the stimulus, so detectability can be parametrized by adjusting the intensity of the cues. Note that detectability is not only dependent on the intensity of the sheer stimulus, but is also mediated by contrast factors (i.e. how easily the stimulus can ‘pop out’ from its contextual surround; [1]), whether the cue is perceptually available (i.e. unoccluded) and the integrity of the sensory organs.

Crucially, attention itself is malleable. Cues that are reliably followed by important outcomes, from the agent's perspective, will tend to capture more attention as if they were physically intense. Conversely, cues that are not good predictors of important outcomes, even if physically intense, tend to lose their attention-capturing potential (e.g. [3]).

This adds a layer of behavioural flexibility that is potentially adaptive in volatile environments, hinting at the biological problem that this mechanism solves. The very existence of behaviour has been justified as an adaptation for active goal pursuit in organisms for whom vital resources (e.g. nutrients) are not stationary and cannot simply be waited for [4]. These organisms must efficiently connect perception to movement to orient towards goals [5,6]. Learning—the capacity for behavioural change—is typically understood as an adaptation to variability across lifespans [7]; for example, individuals born in different environments may benefit from learning subgoals tailored to local conditions. Extending this rationale, mechanisms allowing meta-learning such as dynamic salience can represent adaptations to variability within a lifespan—letting organisms optimize when to learn and when to ignore cues. By increasing associability for predictive cues and down-regulating it once cue–outcome relationships are familiar, dynamic salience allows learning systems to allocate responsiveness or caution as appropriate, adjusting flexibly when the environment affords a learnable structure. For such a mechanism to be adaptive, the environment must contain at least some latent regularities: pure volatility should not give rise to it if patterns cannot, in principle, be revealed. The ‘default’ level of attention capture of a cue is commonly referred to as *unacquired salience*, while the level of attention attributed to prior experience with the cue is termed *acquired salience*.^1^

In summary, the salience of a cue is partially determined by its intrinsic physical properties and its capacity to stand out from its surroundings, while the remainder depends on the subject's prior experience with that cue. Conceptually, the former is a static property of the cue, while the latter is malleable and can increase or decrease depending on the cue's capacity to predict relevant outcomes for the subject. This rationale carries substantial implications, the foremost being that behaviour is meta-malleable. Concretely, behavioural change can be initially influenced by how perceptible a cue is, but subsequent changes in behaviour will depend on changes to that cue's salience. Those latter changes depend on the subject's history of interaction with the cue. By disentangling the components of salience, we clarify its role as an emergent property of the continuous interaction between agent and environment. This paper examines the conceptual challenges of characterizing and measuring salience malleability and draws out its implications for adaptive behaviour, closing with directions for better integrating neural and behavioural findings.

## 2. A brief note on meta-malleability

The ‘meta-’ suffix is used to express recursion. Recursion means that one process or property is applied to itself. The main claim of this article is that behaviour is meta-malleable and that one thing that allows so is dynamic salience. As recursion stacks levels and these are easy to conflate, we need to frame the rationale with a few specifications. Let behaviour be the object of modification and pertain to level 0. Learning implies updating behaviour, so it would be at level 1 of malleability. If the process of learning is itself modifiable by means of salience being dynamic, that would lie at level 2; this implies meta-malleability because dynamic salience updates what can update behaviour. We can go on further with infinite levels, but crucially, what is needed for something to be meta-malleable is having at least two levels of malleability above it. Making something merely malleable requires only one level above it. An additional clarification requires marking a difference between ‘is’ and ‘allows’. What we are claiming *is* meta-malleable is behaviour, while what we are claiming *allows* behavioural meta-malleability is dynamic salience. In that scheme, learning only allows malleability of behaviour; it is a first-order process. So, learning both allows malleability and is malleable—the latter, as noted, by virtue of dynamic salience being able to modify it. Whether learning itself is also meta-malleable (i.e. whether two levels of modification lie above it) is a valid question but beyond the scope of this article (but see §6.2).

The sections that follow instantiate this scheme. Section 3 establishes what behaviour is and how behavioural change (i.e. learning) can be formalized, fixing levels 0 and 1. Sections 4–6 then treat salience as a dynamic quantity that modifies learning trajectories, the modification that lies at level 2, and §7 surveys evidence on how such dynamics are realized in neural tissue.

## 3. Framing behaviour and behavioural malleability

Interpreting behaviour accurately is crucial for understanding the underlying mechanisms involved. To achieve this, we must overcome the external observer bias. From a typical external observer, behaviour is treated as mere output—or, in a slightly better but still inadequate view, as a caused event [8,9]. That stance artificially separates perception from action and misses their dynamic integration. Behaviour is better characterized as closed-loop, whereby movements arise in a coupled agent–environment system as organisms pursue subjective perceptual goals [10,11]. From a mainstream-cognitive-science perspective, salience is typically conceived as an early-stage phenomenon—arising during initial sensory processing and localized in input-dedicated structures (e.g. sensory receptors, primary cortices or unimodal associative regions). By contrast, in a closed-loop or feedback-control view, salience is not solely attributed to early-stage filtering. Rather, it emerges from the interplay of lower-level control loops that include both sensory structures and effector systems—whether overt or covert—that modulate what sensory input is maintained, enhanced or disregarded. In this sense, salience is not simply treated as a stage in the processing pipeline, but as an aspect of lower-order control systems embedded within hierarchically organized agents.

Although behaviour can be understood as a closed-loop process, conventional paradigms for studying behavioural change typically adopt a simplified stimulus→response framework. This approach operationalizes behavioural change as differences in responses measured before and after a controlled experience, essentially treating behaviour as unidirectional rather than dynamically coupled. Researchers favour this framework for practical reasons, as it enables experimental control and clear causal inference. At a minimum, demonstrating behavioural change requires showing that a cue elicits a different output pattern following a researcher-controlled experience (compared to appropriate reference conditions; see [12]). A common method to achieve this is to pair such a cue with a biologically significant or arousing event, a procedure known as Pavlovian conditioning. The goal of this paradigm is to capture, in a laboratory setting, the process by which agents come to engage with useful signals. Once the pairings are complete, the degree to which the agent anticipates the outcome paired with the cue is considered a measure of its theoretical *associative value*. Thus, by conceiving learned behaviour as the response to a previously innocuous cue, Pavlovian conditioning is a tool to attribute behavioural change to experience with associated environmental events. This rationale resembles canonical reinforcement learning algorithms (e.g. [13]); unlike many such algorithms, however, the associative learning framework is agnostic to the affective valence of outcomes, which makes it valuable for describing anticipatory behaviour in both appetitive and aversive settings.

To most people, associative value can be a dauntingly technical term, abstract enough that one loses track of what learning theories are about. This squares with a recent plea to keep sight of the connection between conditioning in the laboratory and learning in natural settings [14]. We therefore establish a recurring example, returned to at several points in this article, that anchors the key concepts originating in laboratory research to behaviour in the wild. Consider a squirrel foraging in a forest. The sound of a ripe fruit dropping to the ground reliably precedes a meal. Such an experience may prompt the squirrel to orient towards the sound the next time it hears it. The sound can then be said to have acquired an appetitive associative value. The *associative* qualifier states that the sound has become linked to the fruit, and the *appetitive* qualifier implies that, because the squirrel values the food, it will approach the sound source. The crack of a branch under a predator's weight comes before an attack and will similarly change the squirrel's behaviour the next time it hears a branch cracking. In this case, though, the squirrel would probably move in the opposite direction, meaning that the sound acquired an *aversive* associative value. This example makes it clear that associative learning concerns how an organism's behaviour changes upon meaningful experiences. The trajectory of those changes varies, however: it can be abrupt or gradual, and it can follow simple or intricate patterns. An accurate understanding of associative learning thus requires formalizing its terms and rules of change.

The associative value of a cue is often quantified by measuring the vigour or likelihood of a response that anticipates the significant outcome upon its presentation. This measurement enables the characterization of the engagement of agents with relatively simple informational regularities in their environment. The trajectory of associative value across structured experiences has been examined through theoretical models^2^ that formalize trial-by-trial behavioural adjustments. Early associative learning models updated the value of a single cue with fixed associability [19]. Second-generation models formally dissociated cue-related and outcome-related associabilities, with the former corresponding to salience; these models also introduced cue-combination rules that allowed negative (inhibitory) values while maintaining cue-specific but fixed salience parameters [20]. This framework sets the stage for models in which salience itself is learned, that is, in which associability becomes dynamic. In the following, we detail models that incorporate salience into the learning process, including both those that treat it as fixed and those that allow it to change dynamically.

At first, on each occasion the cue and the outcome are presented in sequence, known as a trial, the associative value of the cue increases.^3^ This process is revealed by an increase in the probability or vigour of the anticipatory response (measured through behavioural, peripheral or neural indices). However, empirical observations support that these gains in associative value do not continue indefinitely, but rather grow by increasingly smaller steps—the acquired importance assigned to the cue reaches an asymptote in a negatively accelerated fashion [24]. The Rescorla–Wagner model [20] is an ingenious formalization of these stepwise changes in behaviour, where the amplitude of change decreases with each paired presentation of cues and outcomes (or their omission). It states that

(3.1) $$V_{i+1}^{X}=V_{i}^{X}+\Delta V_{i}^{X},$$

meaning that the associative value, V, of a cue, X, for the next trial, i+1, is determined by the current value, $V_{i}^{X}$, of that cue plus a gain term, $\Delta V_{i}^{X}$. This gain term for one trial would, in turn, be determined by

(3.2) $$\Delta V_{i}^{X}=\alpha_{i}^{X}\times \beta_{i}^{\lambda }\times \left(\lambda -\sum V_{i}^{N}\right),$$

where the expression in parentheses represents a prediction error for the current trial; that is, the discrepancy between the expected outcome, $\sum V_{i}^{N}$ (explained below), and the experienced outcome, λ, usually coded in binary (1 for presence, 0 for absence of the outcome).

The outcome expectancy is given by the sum of associative values $V_{i}^{N}$ for all cues N that are present during a trial i, that is, $\sum V_{i}^{N}$. The prediction error will influence the gain value, $\Delta V_{i}^{X}$, with a modulatory permissive role for the rate parameters $\alpha_{i}^{X}$ and $\beta_{i}^{\lambda }$. The parameter β represents the associability of cue–outcome pairings attributable to outcome-related factors (e.g. habituation, motivational dispositions), λ, while the parameter α represents the associability attributable to cue-related factors. The latter has been linked to the concept of salience, which—as mentioned above—can be further partitioned into unacquired and acquired components, which is the focus of this paper. Essentially, salience is not a property of the cue in isolation nor solely of the subject, but emerges from their interaction. The parameter α thus reflects both cue-driven features (e.g. intensity, complexity) and subject-dependent variables (e.g. perceptual integrity, attentional history). When data on the trajectory of associative strength across trials are available, theoretical learning curves can be fitted to empirical values by leaving one of the components of the learning rate (i.e. α or β) as a free parameter. The resulting values of the optimized free parameters fitted to the model may vary between subjects and between experimental conditions.

In the former case, the obtained values can serve as an index of pre-existing individual differences, while in the latter, they help infer the influence of some tractable external factor (e.g. cue detectability, deprivation, ablation, inactivation, drugs) on learning. Next, we describe how the role of salience can be empirically demonstrated and illustrate how both unacquired and acquired salience have been employed to account for various learning phenomena.

## 4. Methodological and theoretical implications of cue salience

### 4.1. Infotactic behaviour as a proxy for cue salience

Salience is commonly defined in terms of the attention commanded by a cue. Conceiving attention as a subjective or covert process inherently complicates its detection and quantification,^4^ since such processes cannot be directly observed and must instead be inferred from proxy measures. Nevertheless, attention—and, by extension, salience—can be operationalized by the degree to which agents orient their sensory systems towards a cue. Orienting towards some cue source can be attributed to different factors. Cues associated with rewards are known to ‘pull’ perception towards them, a robust phenomenon known as value-modulated attentional capture (VMAC; [27]). Under certain conditions, however, organisms have been observed to ‘work’ for pure information [28,29], a tendency known as infotaxis [30] or epistemic action [31]. For orientation behaviours to count as infotactic, VMAC confounds should be ruled out, narrowing the set to actions aimed at information gathering. Such behaviours may lack immediate utility (in reward or threat terms) but can still support pragmatic goals over longer time scales. We return to the tension between these factors in §6.1, but first, we present a few empirical demonstrations of infotaxis.

For instance, Kaye & Pearce [32] noted that when a light was turned on in a conditioning chamber, untrained rats (*Rattus norvegicus*) reared up or touched the bulb with their snouts or paws. Both of these behaviours involve directing their visual receptors towards the brightest area of the chamber, which is the zone with the highest contrast between the on and off states of the light bulb. The fact that rats exhibit orientation tendencies despite not being trained to do so led the authors to consider these responses a proxy of the unacquired salience of the cue. Moreover, as mentioned before, this behavioural tendency decreased with repeated presentations of light alone, showing that the unacquired salience fades when a cue fails to predict significant outcomes. This demonstrates that salience is not a fixed sensory property, but a dynamic attribute that can wane through repetition, even when sensory access to the cue is fully guaranteed.

Prominent models of salience computation (e.g. [1]; for a comprehensive review, see [33]) propose mechanisms to extract information from visual cues by selecting relevant features in a given scenario. Importantly, the way in which animals extract visual information depends on their body plan. Animals with little eye or neck mobility, like most fish, depend on whole-body orientation. Many birds (e.g. owls) have very limited eye mobility, and some rely heavily on head saccades that depend on neck movements (e.g. pigeons; [34]). Yet, there are animals capable of tracking or collecting visual information using ocular saccades, such as primates, cats and chameleons [35]. A non-tetrapod example of eye saccades without neck mobility is the archerfish [36], which must combine them with whole-body orientation. This gradient constitutes a progressive reduction of the energetic cost and predation exposure of visual exploration [37]. However, although whole-body locomotion and saccades differ in sensory mechanics—approach changes the projection of the retinal cue, while a saccade shifts the gaze without changing the distance—both behaviours typically instantiate the same control process: reducing perceptual uncertainty by directing receptors towards information-rich regions of the environment. Crucially, cues from sensory modalities other than vision evoke distinct orienting responses. For example, Holland [38] found that auditory cues typically evoke a startle or head-jerk response, rather than approach behaviours directed at the source of the cue, among untrained rats. According to this author, orienting behaviours to different cue modalities are influenced by the physical characteristics of the stimuli; specifically, the structure of these responses may maximize the ‘receipt’ of the cue by the agents’ sensors. In Holland's study, orienting responses to the auditory cue decreased with its repeated presentation alone, further underscoring how novelty modulates salience. Notably, in the studies by Kaye & Pearce [32] and Holland [38], orienting responses to auditory and visual cues increased when paired with food. This can be explained by the cues gaining additional salience as signals of a relevant outcome. Overall, these studies’ results support the idea that infotactic tendencies encompass both unacquired and acquired components of salience.

Although initially foundational, relying on orienting responses as a proxy for salience may sometimes pose methodological challenges. For example, when cues from different modalities are compounded (presented concurrently), the specific orienting responses for each may compete with one another for control. This competition could hinder the ability to isolate the salience of both the compound cue and the individual cues. Similarly, when a target cue signals an affectively loaded outcome (appetitive or aversive), the anticipatory response to that outcome may compete with the otherwise expressed orienting response. Consequently, alternative methodological approaches have been adopted when inquiring about salience.

The findings described here illustrate how orienting or infotactic behaviours can serve as measurable indicators of salience, capturing both its unacquired and acquired components. These behaviours offer an initial, though constrained, means of operationalizing salience. The next section introduces a more sophisticated—and ultimately more productive—framework in which associability, rather than orienting behaviours, serves as the basis for inferring salience.

### 4.2. Inferring salience from associability

If an agent pays attention to a cue, it will more likely associate that cue with a significant outcome. Thus, an alternative way to infer salience, α, is by the amount of associative change from one cue–outcome pairing to the next presentation of the target cue that is not attributable to the prediction error or to outcome-related factors, β. Assuming that cues A and B have equivalent associative values (ideally, a nominal zero; $V_{i}^{A},V_{i}^{B}\approx 0$) in a trial in which both A and B are paired with the same outcome under reasonably similar conditions, a discrepancy in anticipatory response upon a subsequent independent presentation of A and B would indicate pre-existing salience differences ($\alpha_{0}^{A}\neq \alpha_{0}^{B}$). Note that this approach enables the evaluation of salience in both elemental (A+ / B+)^5^ and compound (AB+) cues.

### 4.3. Partitioning unacquired and acquired salience

The dissociation between unacquired and acquired^6^ salience can be succinctly formalized as

(4.1) $$\alpha^{X}=\phi^{X}+\varepsilon^{X},$$

where ϕ denotes unacquired salience (influenced by sensory attributes) and ε denotes acquired salience (shaped by prior experience) of a given cue, *X*. Once partitioning the components of salience, what follows is to identify the conditions under which acquired salience changes. Early models addressed this issue by positing that gains in acquired salience are proportional to (i) the prediction error in the latest trial (i.e. *surprisal*; [40]), (ii) the degree to which the target cue yields a consistent outcome (i.e. *predictiveness*; [42]) or (iii) a combination of both (e.g. [43,44]). Although many hybrid models, appropriately parametrized, do a good job of recreating key associative phenomena, a recent simulation study reported that the Esber–Haselgrove model outperformed its predecessors [45]. We therefore adopt this model in the subsequent analysis, beginning with an account of how it works. Esber & Haselgrove's [39] model proposed that the acquired salience of a given cue is proportional to its current status as a predictor of significant outcomes. Therefore,

(4.2) $$\varepsilon^{X}\propto \sum V^{X\to O},$$

meaning that the acquired salience of a cue, *X*, derives from the sum of associative strengths, V, corresponding to all significant outcomes, O, with which that cue has been paired. Here, the rightward-pointing arrow indicates a temporal sequence in which the cue precedes the outcome, not a mathematical mapping. In short, it is assumed that distinct outcomes independently contribute to the salience of a cue. Moreover, Esber & Haselgrove [39] posited that the absence of an expected outcome (henceforth termed ‘no-outcome’, for brevity) constitutes an emotionally potent outcome itself (see also [46] and [47]) and, correspondingly, should be included in $\sum V^{X\to O}$. In a scenario where a single outcome may follow the cue, *X*, the acquired salience is determined by how well the cue has predicted the outcome, as well as the extent to which the cue has led to no-outcome when it was expected in the past. Thus,

(4.3) $$\varepsilon^{X}\propto V^{X}+{\bar{V}}^{X},$$

where $\bar{V}$ represents the value of the no-outcome associative component. To include this component as a determinant of acquired salience, the Esber–Haselgrove model deviated from the Rescorla–Wagner delta rule ΔV, instead incorporating the Pearce–Hall model's delta rule [40], which determines gains in excitatory and inhibitory associative strengths separately:

(4.4) $$\Delta V_{i}^{X}=\alpha_{i}^{X}\times \beta_{i}^{\lambda }\times \left(\lambda -\left(\sum V_{i}^{N}-\sum {\bar{V}}_{i}^{N}\right)\right).$$

The gain in excitatory associative strength for a cue in each trial, $\Delta V_{i}^{X}$, is given by the discrepancy between the outcome encountered, λ, and the overall prediction of this outcome—which encompasses both positive $\sum V_{i}^{N}$ and negative $\sum {\bar{V}}_{i}^{N}$ expectations antagonizing each other. Conversely, the gain in inhibitory associative strength is determined by

(4.5) $$\Delta {\bar{V}}_{i}^{X}=\alpha_{i}^{X}\times \beta_{i}^{\lambda }\times \left(\left(\sum V_{i}^{N}-\sum {\bar{V}}_{i}^{N}\right)-\lambda \right),$$

where the terms in the outermost parenthetical subtraction are reversed, implying that inhibitory gains are positive when the cue is followed by a surprising absence (or reduction) of the outcome and negative when it is followed by surprising encounters of the target outcome. Equations (4.4) and (4.5) are restricted to produce positive gains. Therefore, if the result of the outer parenthetical term is less than zero, both components of this system of equations are rendered ineffective.

Note that unlike acquired salience $\varepsilon^{X}$, which is given by the aggregation of excitatory and inhibitory associative strengths of the cue, the overt anticipation of outcomes can be excitatory or inhibitory depending on the subtractive relationship between those strengths. Then, outcome anticipation will manifest whenever $V>\bar{V}$ and, conversely, no-outcome anticipation will manifest whenever $V<\bar{V}$ [40].

A final addition to Esber & Haselgrove's modelling of acquired salience is that the salience decreases related to novelty loss are computed separately from the dynamics related to associative strength. As described in the section above, repeated presentations of a cue without being followed by any explicit outcome progressively attenuate orienting responses towards it, but also its associability—both being indices of salience. Note that this decline does not constitute extinction, as that phenomenon presupposes prior conditioning; it simply reflects the loss of unacquired salience through repeated exposure. Below, we describe how Esber & Haselgrove [39] incorporated this process into their model of dynamic salience.

Esber & Haselgrove [39] proposed that the salience of a repeatedly presented cue decreases when its occurrence becomes predictable from other features of the environment, including both discrete cues and the general context (cf. [48]). Thus, the complete computation of salience—hereafter *effective salience*—is given by

(4.6) $$\alpha^{X}=\phi^{X}+\varepsilon^{X}-k\times \sum V^{\text{pre}\to X},$$

where $\sum V^{\text{pre}\to X}$ represents the expected degree to which the cue X is predicted by the preceding environmental features, with the gains calculated using an analogous delta rule. The constant k, bounded between 0 and 1, weights the predictive term $\sum V^{\text{pre}\to X}$; it thereby determines how much pre-exposure experience can subtract from unacquired salience. A value of k=1 would make the maximum possible loss virtually equal to the cue's full unacquired salience, which seems implausible, so values of k<1 are reasonable.

## 5. Empirical phenomena explained by multi-faceted, dynamic salience

### 5.1. Static salience modulates learning rates; dynamic salience modulates learning-curve shapes

A basic prediction regarding unacquired salience is that, when other factors are held constant, it should modulate the pace of learning curves. This has typically been shown in non-human animal classical conditioning studies [49–51] and in a few human studies (e.g. [52–54]; see [55], for a comprehensive review). Models with fixed salience parameters, such as the Rescorla–Wagner model [20], assume constant learning rates for each cue. Within this framework, cues with greater unacquired salience—that is, higher fixed salience values—are predicted to produce faster learning curves, without differences in their shape or asymptote. That is, learning curves for both weak and strong cues will approach the value of λ in a negatively accelerated fashion, albeit at different rates (see fig. 2 in [56]). On the other hand, models that incorporate dynamic salience modules, such as Esber & Haselgrove's [39] model, predict that the shape of the learning curve will differ according to the cue's unacquired (i.e. ϕ) salience. This prediction arises from the joint contribution of the modules in the system of equations in the Esber–Haselgrove model presented earlier, rather than from any single expression in isolation. The contrast between fixed and dynamic salience assumptions is essential as only models including the latter can reproduce differences in the shape of the curve.

**Figure 1.**
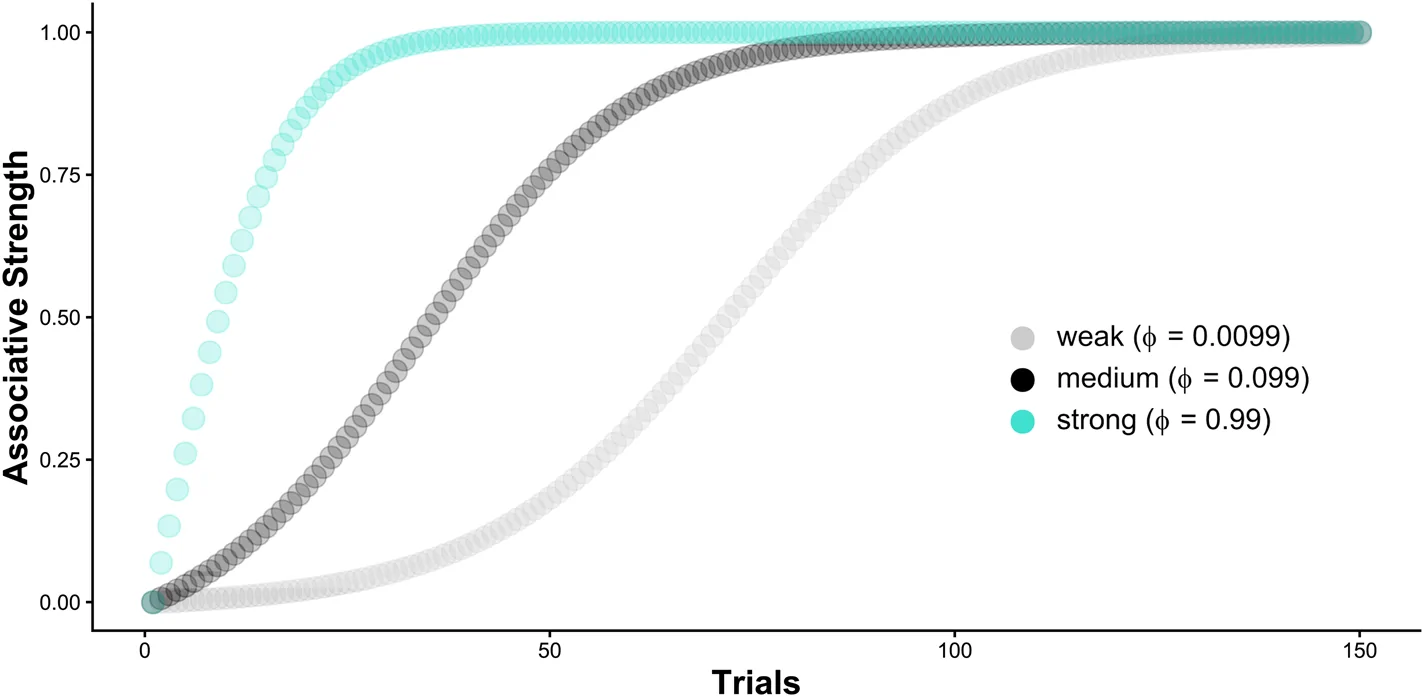
Different levels of unacquired salience produce varying shapes in learning curves according to the Esber–Haselgrove dynamic salience model. *Note:* The parameter $\beta^{X}$ was set to 0.7. The script used to produce the data in this figure is available at http://github.com/RSosa-BehavioralScience/dynamic_salience.

As seen in figure 1, a very high value for unacquired salience results in a predominantly negatively accelerated learning curve, while lower values of this parameter produce S-shaped learning curves. The latter two-faceted acceleration in learning curves has been reported in the literature (see [57]), providing a predictive advantage for dynamic salience models. However, it remains to be examined whether low-intensity cues systematically produce S-shaped rather than negatively accelerated learning curves and whether this predictive advantage persists despite the reduced parsimony introduced by additional parameters and/or update rules.

While the curves shown in figure 1 derive from simulations rather than empirical data, they illustrate how different fixed values of unacquired salience modulate the shape of acquisition curves under a dynamic salience framework. However, inferring unacquired salience—or other modulatory factors—from learning curves can be difficult (see [58,59]). In this context, conditioned responding may soon be influenced by the rate associated with acquired salience itself, making it hard to isolate the contribution of the unacquired component. In addition, a nonlinear mapping between unacquired salience and behavioural updating would complicate matters even further [60]. A more conservative diagnostic would rely on one-shot learning (see [61])—that is, the response observed upon the first exposure to a cue–outcome pairing that has never been experienced before. This method would unconfound acquired from unacquired salience, providing an uncontaminated expression of the latter, since subsequent trials would already reflect the interaction of both factors. Differences in conditioning under one-shot learning would in principle be diagnostic of unacquired salience, because the first exposure to a novel cue–outcome pairing reflects salience before any acquired salience component can contribute. Of note, such a test would be statistically inefficient, given the low power of inference from a single data point, but our claim is that it would be diagnostic in principle.

### 5.2. Low unacquired salience leaves cues at a conditioning disadvantage against concurrent cues

Returning to our foraging squirrel, consider a tree with a distinctive shape that often has fruit beneath it and, in a certain season, gives off a distinctive odour. Because the odour is more easily perceived (i.e. more salient), the squirrel comes to track fallen fruit by the odour rather than the shape. When the same squirrel later encounters the tree outside the odour-producing season, with the shape present but no odour, it fails to recognize it as a food source. This is an instance of *overshadowing*. Overshadowing, a well-known associative phenomenon, occurs when the less intense element of a compound stimulus supports impoverished conditioning when the compound is paired with an outcome (cf. [62]). The Rescorla–Wagner model, as well as other theories based on prediction errors, accounts for this finding by positing that the elements of the compound compete for gains in associative strength. Consequently, the cue with greater salience will accrue associative strength more quickly until the compound reaches the asymptote in λ (see [18]). At this point, the associative strength is split between the competing cues, with an advantage for the most salient cue proportional to its salience differential. Interestingly, salience attenuation through pre-exposure can also promote an overshadowing-like effect [63].

Although overshadowing is a well-established phenomenon, some findings suggest that the mechanism underlying it may not be only that anticipated by the Rescorla–Wagner model. This and similar models predict that overshadowing does not occur in the very first pairing between the compound cue and the outcome because competition occurs only after the cues have accrued some associative strength [18,40]. However, some studies have provided evidence for one-trial overshadowing. For example, James & Wagner [64] found that across three conditioned emotional response experiments, rats showed less stimulus-evoked suppression of licking to either element of a compound than to the single-trial shock-paired compound itself. This leaves open the possibility that overshadowing-like phenomena may instead represent a case of the generalization decrement effect. Such an effect consists of a reduction in an anticipatory response owing to any difference—either from stimulus aggregation or subtraction—between the circumstances in which the cue is trained and those in which its associative strength is tested [18,65,66].

Another lens through which overshadowing can be addressed is by positing that cues compete for gains in salience rather than solely for gains in associative strength ([40], p. 541), and that salience modulates the expression of conditioned responding rather than solely the rate of learning cue–outcome associations. There is some evidence that overshadowing operations impair salience dynamics and not just acquired value. [67] showed that cues pre-exposed in compound (presented together without being followed by an outcome) support better performance in subsequent discrimination training than cues pre-exposed in isolation. This finding suggests that concurrent cues compete for associability updating, not just for accruing associative value (for salience dynamics under pre-exposure operations, see §5.4). The same study showed that canonical excitatory overshadowing left the overshadowed cue with less associability than a control counterpart. Taken together, these results suggest that overshadowing may be influenced by impaired associability (salience) updating in compound cues, provided that associability is allowed to modulate performance and not only learning rate (but note that in standard associative models with salience dynamics, associability modulates only the learning rate; e.g. [39,40]).

Two more unsuccessful predictions of the Rescorla–Wagner model are that (i) cues with similar salience would mutually overshadow each other, and (ii) the overshadowed cue would remain a poor elicitor of anticipatory responses. However, there is evidence that mutual overshadowing occurs with competing cues of low but not high initial salience [68]. Similarly, it has been reported that after compound-cue conditioning, if the associative value of the higher salience cue is mitigated through extinction operations (further presentations of the cue alone), the putatively overshadowed cue ‘recovers’ its response elicitation capacity (e.g. [69]). Such post-overshadowing recovery indicates that overshadowing is not merely a product of a deficit in associative strength gains. The comparator hypothesis [70] explains this by suggesting that the high excitatory status of the overshadowing cue negatively influences the expression of anticipatory responses to the overshadowed cue in the conditioning context. Thus, the overshadowing cue would mask anticipatory responding to the overshadowed cue unless its linkage to either the outcome, the overshadowed cue or the context is weakened—where the former two are achieved through extinction. In sum, while overshadowing-like phenomena can be accounted for by salience-as-associability accounts, interactions with other mechanisms seem warranted.

### 5.3. Redundant cues support poor expression of anticipatory responses

Let us follow up with the distinctively shaped, season-dependent odour-producing tree. Now, consider a different squirrel that encounters the tree in the odourless season and learns to track its shape, because there is no other salient cue available. Later, when the shape and the odour are present together, this squirrel will still use the shape to track fallen fruit. As a consequence, if it later encounters the odour on an occasion with no shape in sight, it will not track the odour to search for fallen fruit, disregarding it because the shape had already been established as a good predictor of a meal. This shows how a cue of low physical salience, the shape, can come to dominate conditioning over a physically more salient one, the odour, once experience has established the former cue as a reliable predictor of a meal. In associative learning theory, the phenomenon is known as *blocking*. Just as cues with greater unacquired salience can hinder the acquisition of associative strength in coextensive cues conditioned in a compound (i.e. overshadowing), in so-called blocking phenomena, cues with greater associative strength can also yield a similar conditioning deficit. Specifically, if a cue is rendered a good predictor of an outcome, subsequent pairing of this cue and a companion cue with the same outcome will lead to poor anticipatory responses by the companion (see [71]). A common interpretation is that the companion cue provides redundant information and thus does not enter associative gains with the outcome, owing to the lack of surprise in compound–outcome pairings [20].

A different account of blocking invokes salience. According to Mackintosh [42], blocking occurs because the change in a cue's salience depends on its predictive value relative to the other cues present. Specifically, the prediction error generated by each cue in a compound is compared with that of the others, and the cue yielding the smallest prediction error (i.e. the best predictor of the outcome) receives the larger salience gain. The two mechanisms are not mutually exclusive, however (see [66]). Regardless of the underlying mechanism, blocking phenomena underscore the importance of prior experience in new learning. Even when experiencing identical cue–outcome pairings, the learning paths will diverge for agents with differing learning histories. Nevertheless, recent evidence suggests that blocking is not as straightforward as previously thought.

Maes *et al.* [72] reported a series of 15 failures to reproduce the blocking effect in different laboratories, model species (mice and several rat strains) and the affective valence of the outcome element in compound–outcome associations, measuring conditioned suppression or magazine approach to the target cue. Moreover, a Bayesian meta-analysis of this series of experiments yielded substantial evidence in favour of the null hypothesis (i.e. no blocking effect). The authors suggested that blocking might be an elusive and parameter-dependent phenomenon, unlike the previous notion of it being a highly reproducible effect. Some putative culprits of the previous neglect of this nuance, according to these authors, are the selection of liberal control conditions, a file-drawer effect and publication bias leading to an overabundance of positive results in the literature. In response to this report, Soto [73] argued that most of the results in the series of experiments conducted by Maes *et al.* [72] could be accounted for by principles firmly rooted in some associative learning theories. Specifically, replication failures could have arisen from floor effects, the same modality of elements in the compound cue and differences in salience between the blocking and blocked cues. Regarding this latter aspect, Soto [73] asserted that tones tend to be more inherently salient than lights for rodents, making the blocking of lights by tones difficult to achieve in a subset of the experiments reported by Maes *et al.* [72]. Whether failures to replicate blocking-like effects were owing to an issue with the difficulty of detection or true impossibility, what seems uncontroversial is that, as acknowledged by Maes *et al.* [74], unacquired salience is probably a moderator of blocking. This highlights the importance of interactions between unacquired and acquired salience, as will be discussed in the last part of the next major section.

Blocking-like phenomena have been studied and theorized about almost exclusively within the associative-learning tradition. As predicted by Sosa & Alcalá [8, p. 17], active inference theorists—a line of inquiry showing intense growth—have recently weighed in to advance the conceptualization of conditioning phenomena such as blocking. Anokhin *et al.* [75] proposed that the goal of enacting behaviours is to minimize the expected surprise of future perceptual states. They argued that, for an agent to anticipate an outcome upon cue presentation, it must first identify those cues by orienting or attending to them. Importantly, in this framework, attention is regarded as a covert action serving the same functional role as overt perceptual-orienting behaviours such as saccadic eye movements (see also [8]).

To provide a relevant proof of concept, Anokhin *et al.* [75] simulated a scenario in which an agent had to select one of two directions to obtain a reward and avoid an aversive stimulus. The only state signalling which direction to take was the experience of a cue just before the decision point. However, the only way to experience that cue was to take a slight detour on the path towards the decision point. Under these circumstances, the agent consistently took the detour owing to the informative value of the cue (i.e. it helped minimize surprise). Crucially, when an additional cue with the same signalling value was introduced, the agents did not orient towards it because reaching the first cue had already resolved uncertainty, thus nullifying the epistemic value of the second cue. The interpretation of this result was that the artificial agent had learned an epistemic habit that blocked the acquisition of new associations. Thus, blocking occurred not merely because of the affective value acquired by the first-learned cue or its sheer salience, but because of the competition between potential epistemic actions (i.e. attending to the first versus the second cue). Because the authors equated overt perceptual orientation with covert attention, this *in silico* experiment directly translates to blocking in conventional Pavlovian-conditioning paradigms. Such alternative explanations are valuable because they enrich algorithmic approaches by presenting acquired salience not as a passive property but as a consequence of agents’ own actions, whether overt or covert.

### 5.4. Associability—not just orienting tendencies—wanes with repeated exposure

In our squirrel example, cues that reliably come before important events, such as finding a meal or being attacked by a predator, become both a trigger of certain behaviours and attended to. The sound of wind moving the leaves, by contrast, is not followed by anything of consequence; the squirrel has heard it countless times with nothing ever coming after it, so it comes to treat the sound as background, allocating little attention to it even though it is perfectly perceptible. If, for example, an aerial predator's approach later produces that same sound of moving leaves, the squirrel would probably be slow to learn that the sound is now important. Known as *latent inhibition*, this instantiates how repeated prior experience with a cue that was not followed by any important outcome can leave it at a learning disadvantage. Latent inhibition involves repeatedly presenting an affectively neutral cue without a subsequent outcome, resulting in a decreased learning rate to that cue [76]. This phenomenon is important because it aligns with observations that describe a reduction in the ability of a neutral cue to elicit an orientation response through repeated exposure. Thus, both orienting responses (i.e. infotactic tendencies) and associability can be considered behavioural proxies for the otherwise covert construct of ‘salience’. Moreover, the waning of salience from pre-exposure demonstrates that salience can be altered even without the modulation of explicit affectively significant outcomes.

One of the most consistent empirical findings regarding latent inhibition is the modulatory role of the relationship between the target cue and the context in which it is pre-exposed [48,77]. This regularity was first documented in habituation phenomena (although evidence is mixed; [78,79]) and later observed in latent inhibition, establishing a theoretical link between the two processes. In both cases, when the context preceding an affectively significant outcome or a neutral cue comes to reliably predict its presentation, responding to that cue—whether overt or covert^7^—becomes reduced (but see [80], for a more nuanced scenario).

**Figure 2.**
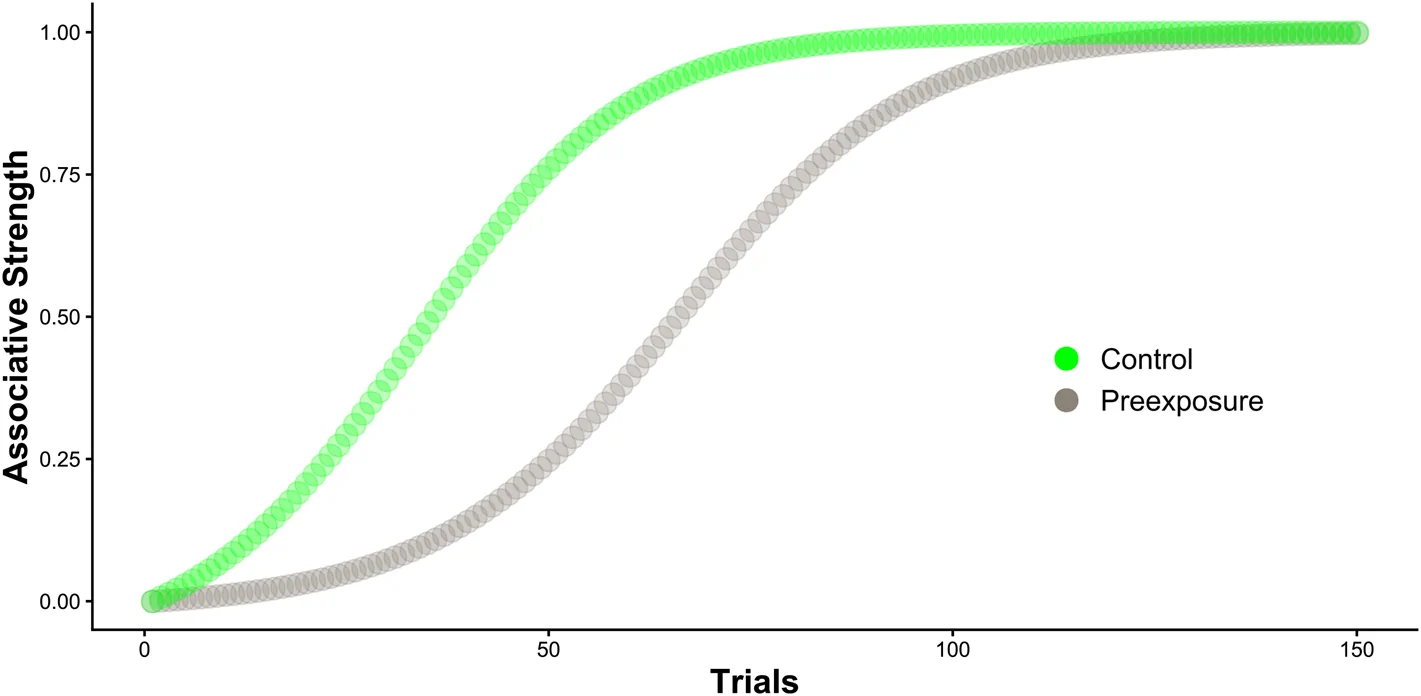
Slower acquisition of excitatory associative strength by a pre-exposed cue according to the Esber–Haselgrove model. *Note:* The simulations used 200 pre-exposure trials, unacquired salience (ϕ = 0.1) was set equally for both cues, and k to 0.99. Note that pre-exposure has a similar effect on acquisition of inhibitory associative strength (not shown). The script used to produce the data in this figure is available at http://github.com/RSosa-BehavioralScience/dynamic_salience.

Importantly, not only is the static general context capable of exerting this influence over responding to pre-exposed cues. For example, Honey & Good [81] reported that a discrete cue reliably predicting the presence of another cue decreased the orienting responses otherwise elicited by it. This finding supports the assumption that the salience of a cue is modulated by its predictability (or surprisal), which is not inherent to the cue itself but is relational to the context in which the cue is embedded. In the Esber–Haselgrove model, this connection to the context is represented by the $\sum V^{\text{pre}\to X}$ component of equation (4.6). In our implementation of the model, $\sum V^{\text{pre}\to X}$ grows asymptotically towards $\phi_{X}$, thus preventing negative values of effective salience $\alpha_{X}$ (refer to equation (6.1)). As figure 2 shows, cue pre-exposure produces a latent inhibition effect, dampening salience and slowing the acquisition of associative strength, as if the pre-exposure cue had lower physical intensity (see figure 1).

**Figure 3.**
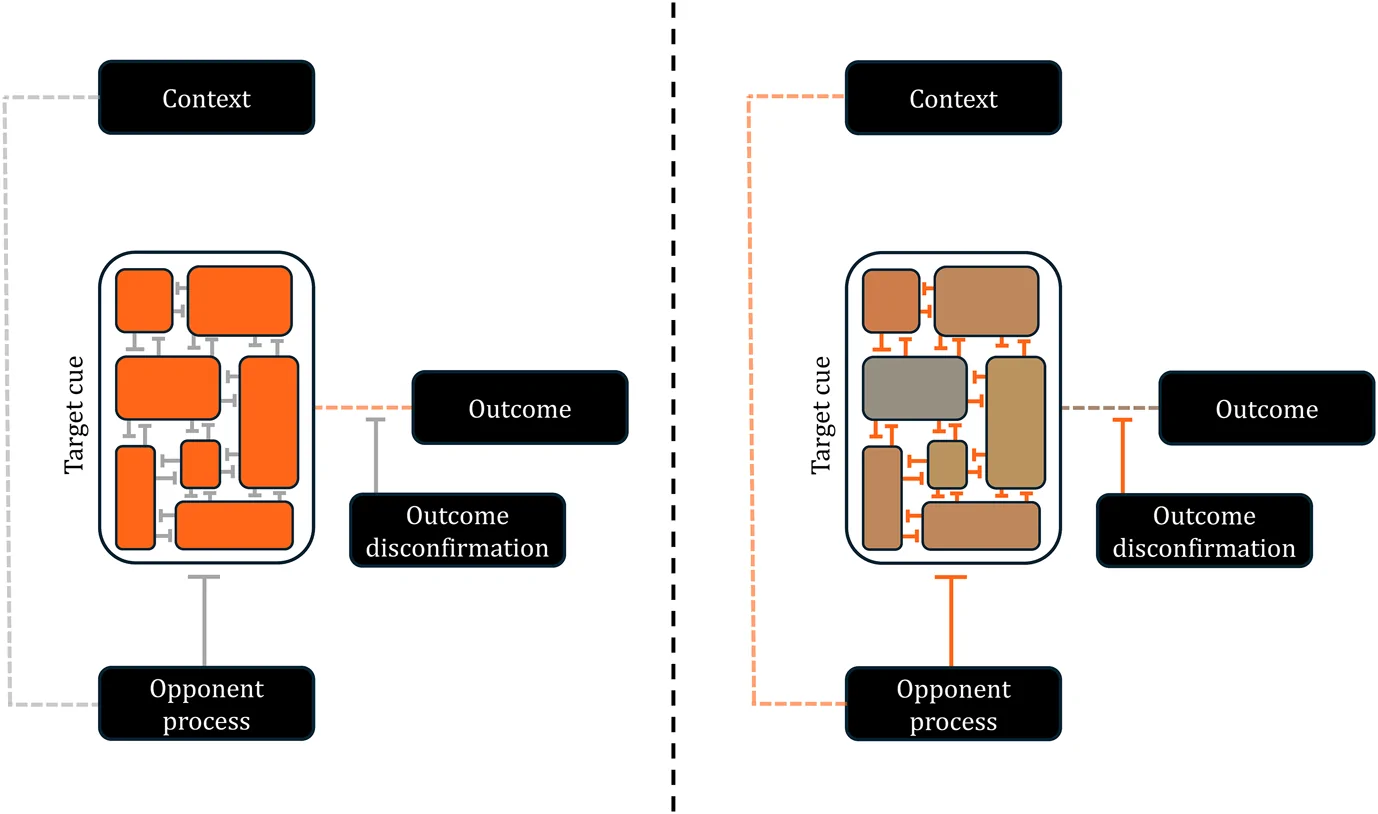
Possible mechanisms leading to latent-inhibition-like effects. *Note:* the left-hand panel shows the state of the system before pre-exposure of the target cue, while the right-hand panel shows the state after pre-exposure. Elements within the large white rectangle represent subunits composing the target cue, and T-shaped lines represent inhibitory connections. Brick red components of the diagram indicate active status, grey components indicate inactive status and greyish components indicate inhibited status. These mechanisms are not necessarily mutually exclusive.

Pre-exposure-related salience loss has been explained by at least three distinct mechanisms, only one of which strictly requires the context to predict the cue; all have been invoked to explain both habituation and latent inhibition. According to Schull [82], any stimulus—whether affectively loaded or not—evokes a subsequent opponent process that, upon repeated presentation, emerges earlier and mitigates both its internal processing and the associated behavioural output. This mechanism (i.e. opponent anticipatory activity) would help agents counteract environmental disturbances and maintain equilibrium (see also [83] and [84]). Alternatively, McLaren & Mackintosh [85] proposed that the sensory properties of a cue can be divided into constituent elements that, with repeated exposure, become predictive of one another and mutually inhibitory. Finally, Hall & Rodriguez [86] proposed that novel—supposedly neutral—cues may carry some expectancy that an affectively loaded outcome will follow. The disconfirmation of that outcome through repeated non-occurrence builds an inhibitory state capable of suppressing subsequent excitatory behavioural tendencies. Note that this latter mechanism does not affect salience alone, but rather involves non-specific inhibitory forces directed towards affective events. Figure 3 illustrates these hypothetical mechanisms.

## 6. Further considerations about salience and its malleability

### 6.1. Trade-offs between infotactic tendencies and affective valence

Agents are equipped with behavioural systems to meet their needs and avoid harm, but these survival mechanisms can sometimes be overridden by information-seeking behaviour. This behavioural pattern has been reported across mammals and birds (see [87]). Goldfish (*Carassius auratus*), by contrast, track rewards but are not lured by information for its own sake [88]. One possibility is that information-seeking of this kind depends on the more elaborated pallium (homologous to the mammalian cortex and white matter originating therein) found in tetrapods (land vertebrates and their descendants), a structure far less developed in ray-finned (*actinopterygian*) fishes. The usual explanation for this is that infotactic behavioural tendencies help disambiguate the actual state of the world (e.g. [89]). However, reducing uncertainty always competes with other valued activities available to the organism. For example, Ajuwon *et al.* [90] found that rats strongly prefer an informative option when presented with two equally probabilistic rewards, even if the information could not be used to enhance reward intake. Yet in this experiment ‘good news’ (signals of impending reward) was pursued more easily than ‘bad news’ (signals of impending no-reward), although both were equally informative. This suggests that both the amount of information and its affective valence influence information-seeking behavioural tendencies.

The observation that organisms are willing to work for news, and more so for good news, aligns with [91] Law of Effect in a broad sense. A literal interpretation, in which behaviours recur because they are followed by a satisfying outcome (i.e. reducing a need), does not readily accommodate information-seeking that yields no increase in reward [90]. The tension is resolved if predictive cues acquire rewarding value through their information status and Pavlovian relationship to reward, so that the effective consequence is a secondary or conditioned rather than a primary reward. Information-seeking and value-seeking then extend the Law of Effect rather than contradicting it, and the greater pull of good news over equally informative bad news follows from the valence of the predicted outcome. This extension is very much needed to explain behaviours that are not immediately followed by a need-reduction consequence.

To certify the pre-eminence of information-seeking over hedonic pursuit, it is necessary to truly pit them against each other. Le Pelley *et al.* [92] found that cues signalling impending reward captured the gaze of human participants, despite attending to those cues cancelling reward delivery. This finding instantiates the conflict between basic reward pursuing and information-seeking; however, as the study by Ajuwon *et al.* [90] showed, this conflict can be explained by both the affective value and informative value of cues. A clearer demonstration of the trade-off between information and hedonic properties was provided by a study conducted by Koenig *et al.* [93]. In this study, cues signalling impending electric shocks captured the gaze of human participants, demonstrating that under some circumstances, agents orient their attention towards informative cues even when they are aversively loaded.

Framed as ‘costly curiosity’, this urge for information at the expense of hedonic experiences challenges optimal foraging and economic utility accounts of behaviour [94,95]. In suboptimal choice paradigms, organisms are presented with probabilistic options that differ regarding reward density, where the leaner option is informative. In many cases, subjects choose the informative option to a greater extent than the richer one, even though it lowers overall food intake [96]. This phenomenon has sometimes been theoretically addressed by appealing to the increased secondary-reward value of good news (e.g. [97]) and sometimes by the decreased no-reward aversive value of bad news [98]. Yet, a complex synergistic interplay between reward and no-reward signals has also been hypothesized to explain suboptimal choice [99]. Finally, salience has also been invoked to explain this phenomenon [100].

While in laboratory suboptimal choice paradigms information gains are explicitly separated from utility, making information-seeking appear maladaptive, in natural settings things could be different. Suppose our squirrel has two patches to search for food, one with more food but located near a waterfall and the other with less food but lying away from it. The waterfall's constant roar masks the very acoustic cues the squirrel relies on. If a fruit drops to the ground, it cannot hear it and gather it readily. If a predator approaches out of sight, by the time it becomes visible, it could be too late to escape smoothly. So, it would be reasonable for the animal to avoid foraging near the waterfall, even though that means passing up the richer patch, because the location degrades the information it can gather.

So far, we have described solid evidence that information-seeking can, under certain conditions, override activities that would otherwise meet an organism's needs. There is another facet of information-seeking, though. The empirical cases described above instantiate orienting responses that resolve uncertainty. However, uncertainty itself can also attract attention, presumably because it affords a position from which an organism might later resolve that uncertainty by finding hidden invariants. This phenomenon, termed uncertainty-modulated attentional capture, has recently been demonstrated across three experiments that progressively ruled out expected value and association with the best outcome as explanations.

Pearson *et al.* [101] showed that human participants trying to visually fixate a target cue were lured by a distractor associated with both reward and non-reward. The pull this distractor exerted was stronger than that of a cue with less outcome variance (i.e. associated with two smaller rewards of equivalent average utility). This result held even when the low-variance cue had higher average utility than the uncertain cue, and when the maximum value was matched across the two. The authors interpreted this as compatible with models in which salience tracks prediction error (e.g. [39,43]). Specifically, the additive contribution of reward and no-reward experiences to salience, formalized in equation (4.3), offers a natural explanation for why a high-variance cue accrues priority. Pearson *et al.* [101] noted, however, that it is at odds with the conception of information-seeking as orienting towards states that resolve uncertainty. Their participants oriented towards a cue that did not resolve uncertainty but could do so prospectively in natural settings. To reconcile this with findings supporting the uncertainty-resolving variety of infotaxis, Pearson *et al.* [101] invoked a distinction between volitional and automatic attentional capture. They observed that information-resolution effects tend to appear in tasks where orienting to the cue was instructed, which they took to suggest that those effects may be volitional, in contrast to the automatic capture in their own task.

We can map the uncertainty-resolving attentional capture onto our squirrel in the forest example. Suppose that the squirrel encounters a large fruit of a kind that is sometimes ripe and edible and sometimes hollow and empty, making a sound that is sometimes followed by a meal and sometimes by nothing. Because its predictive value is unresolved, it draws enhanced attention. The Esber–Haselgrove model explains that this enhanced acquired salience stems from the fact that the sound is followed by two important outcomes—meal and no meal—whose effects on salience aggregate. Then, the squirrel might orient to the source more readily, inspecting it more closely. This behaviour may yield information that disambiguates the outcome. For example, the hollow fruit may bounce more times than a full one, so increased attention would pay off.

### 6.2. Interactions between associability attenuation and motivational states

The attenuation of associability through cue pre-exposure—known as the latent inhibition effect—is sensitive to the current motivational state of agents. In a study with suggestive implications, Killcross & Balleine [102] found that hungry and thirsty rats developed anticipatory behaviours slowly to a cue paired with food pellets when pre-exposed to that cue while hungry. Similarly, subjects were slowly conditioned to a cue paired with water delivery when pre-exposed to that cue while thirsty. Altogether, latent inhibition was strongest when the drive in the pre-exposure stage matched the outcome in the conditioning stage regardless of the drive state during conditioning. This phenomenon is intriguing because when the salience of cues (α) and the arousal value of outcomes (β) are considered orthogonal—as in most models of dynamic associability—one expects them to contribute independently to learning. However, we can build on Esber & Haselgrove's [39] model to account for the interaction between latent inhibition effects and motivational states. Moreover, this may be realizable in multiple ways.

Recall that the effects of latent inhibition in the Esber–Haselgrove model are achieved by the $k\sum V^{\text{pre}\to X}$ element of effective salience, α, whose gains accrue only when λ = 0. As noted earlier, gains in this term are determined by a standard delta rule and can be set to asymptote in ϕ to prevent negative values of effective salience. Therefore, for canonical latent inhibition, we have

(6.1) $$\Delta V_{i}^{\text{pre}\to X}=r\times (\phi -V_{i}^{\text{pre}\to X}).$$

Here, r is the rate of pre-exposure-mediated decreases in effective salience. To model the modulation of latent inhibition by motivational status, we first need to link these decreases in salience to specific outcomes represented by λ. If we have water and food as outcomes with which the cue X will be associated, these can be represented as $\lambda_{1}$ and $\lambda_{2}$, respectively. The deprivation state for each can be symbolized as $d^{\lambda_{1}}$ and $d^{\lambda_{2}}$, with the former indicating ‘thirst’ and the latter ‘hunger’. Gains in salience attenuation can be modulated by thirst and hunger through the free parameters in equation (6.1). Thus, the motivational status of the agent can modulate either the rate or the asymptote of the effects of pre-exposure. For example,

(6.2) $$\Delta V_{i}^{\text{pre}\to X\lambda_{1}}=r\times \;d^{\lambda_{1}}\times (\phi -V_{i}^{\text{pre}\to X\lambda_{1}})$$

and, alternatively,

(6.3) $$\Delta V_{i}^{\text{pre}\to X\lambda_{1}}=r\times (\phi \times d^{\lambda_{1}}-V_{i}^{\text{pre}\to X\lambda_{1}}).$$

**Figure 4.**
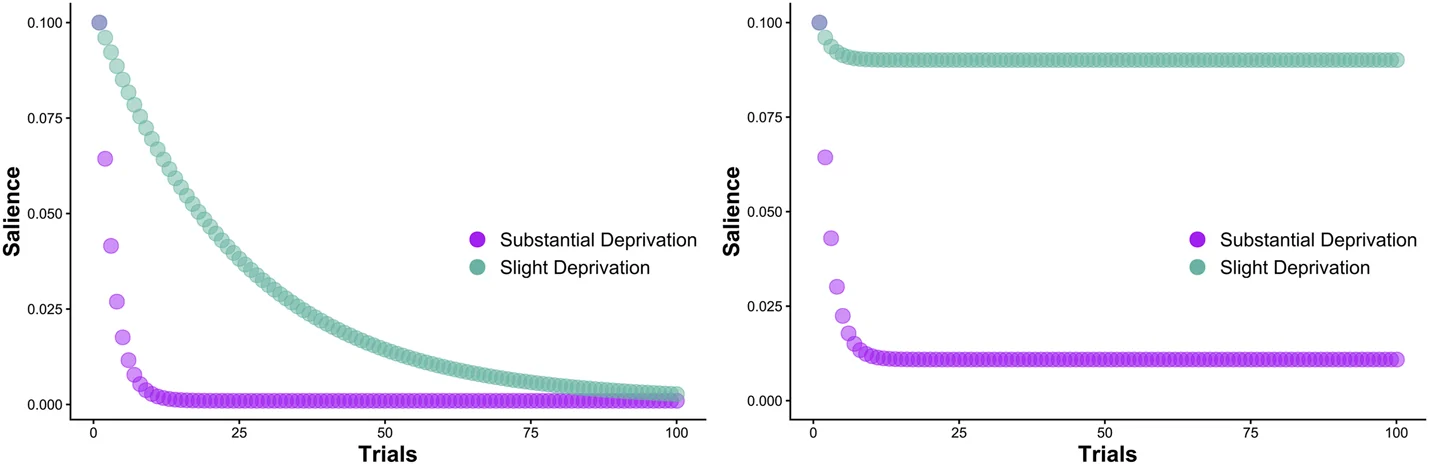
Motivational modulation of associability attenuation by pre-exposure: two alternative implementations illustrating salience decay during substantial and mild motivational states. *Note:* The left-hand panel shows the implementation of the modulatory relationship between motivation and associability attenuation via the rate parameter (see equation (6.2)), whereas the right-hand panel shows the implementation of this phenomenon via the asymptote of pre-exposure effects (see equation (6.3)). Each simulation compares salience loss via pre-exposure across 100 trials in a substantial drive state ($d^{\lambda_{1}}=0.9$) and a mild drive state ($d^{\lambda_{2}}=0.1$). For both simulations, the rate of salience loss, r, was set to 0.4 and ϕ was set to 0.1. The code for these simulations is available in the supplementary material. The script used to produce the data in this figure is available at http://github.com/RSosa-BehavioralScience/dynamic_salience.

In the former case (equation (6.2)), thirst modulates the rate of pre-exposure attenuation of the cue-water associability, whereas in the latter case (equation (6.3)), it sets the maximum possible effect of pre-exposure. Importantly, even if both implementations successfully account for the motivational modulation of cue-pre-exposure effects, each makes different predictions based on the number of cue presentations. Insofar as the interaction in Killcross & Balleine's [102] study reflects a discrepancy in initial salience at the conditioning stage—rather than, say, their ratio—the two implementations diverge. The former predicts that the interaction depends on the number of cue presentations, while the latter predicts it regardless of presentation number. Figure 4 illustrates the implementation of both drive-modulated salience decrease alternatives.

**Figure 5.**
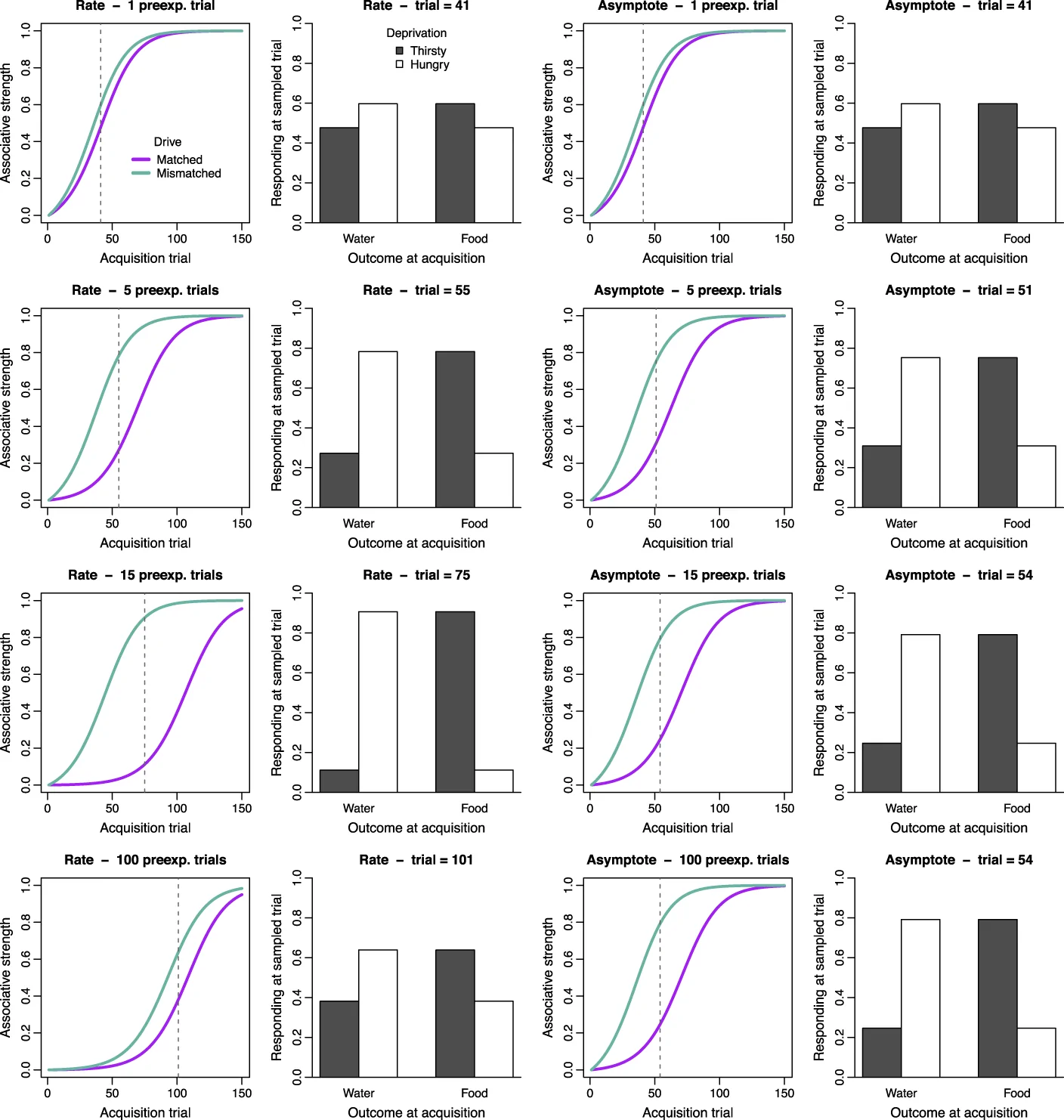
Simulation of Killcross & Balleine's experiment: drive-modulated associability loss under two implementations across pre-exposure length. *Note:* simulated acquisition of conditioned responding to cues for which pre-exposure was applied under a substantial drive state that could match or not match the outcome in conditioning. Columns show the two drive-modulated salience-loss implementations, rate-based (equation (6.2); left) and asymptote-based (equation (6.3); right). Rows correspond to pre-exposure length (1, 5, 15 and 100 trials). For each implementation and pre-exposure length, the line panel plots associative strength across acquisition trials for a cue conditioned with an outcome that matches the pre-exposure drive (Matched) versus one that does not (Mismatched). The dashed vertical line marks the acquisition trial of maximum separation between the two curves, from which the adjacent bar panel is fed; bar panels show simulated responding at that sampled trial, grouped by the outcome paired with the cue at acquisition (Water, Food), with bars within each group denoting deprivation state (Thirsty, Hungry) at the pre-exposure stage. The reversal of relative bar height between the Water and Food groups reproduces the outcome-specific latent inhibition reported by Killcross & Balleine [102]. Pre-exposure parameters (r, ϕ, $\lambda_{1}$ and $\lambda_{2}$) were as in figure 4; the acquisition-phase salience-loss term was weighted by k=0.99. Sampled trial numbers are given in each bar-panel heading. The script used to produce the data in this figure is available at http://github.com/RSosa-BehavioralScience/dynamic_salience.

We simulated the Killcross & Balleine [102] experiment and found the results to depend heavily on three factors: (i) the drive-modulated salience-loss algorithm, (ii) the number of pre-exposure trials and (iii) the window from which data are extracted at the conditioning stage. Figure 5 shows that under the rate-based implementation, the number of pre-exposure trials exerts a non-monotonic influence on the maximum possible difference between drive-matched and mismatched subjects at the conditioning stage. Under the asymptote-based implementation, that influence is monotonic and reaches a plateau relatively early. One difference in measurement worth noting is that Killcross & Balleine's interaction rests on conditioning performance averaged across the acquisition stage, whereas we extracted the maximum possible difference between groups across varying numbers of experienced conditioning trials. In sum, the Esber–Haselgrove model can be expanded to account for drive-dependent salience loss, but the multiple ways the mechanism can be realized yield different predictions that remain to be empirically tested.

### 6.3. Interactions between unacquired and acquired salience

So far, we have discussed findings showing how cue salience updates with experience, and how this modulates subsequent behavioural change as an agent interacts with a given cue. Such an approach considers unacquired salience (ϕ) and acquired salience (ε), two contributors to the associability (α), separately. Tightly controlled paradigms typically hold unacquired salience constant across training, which has made it reasonable to treat it as separable from acquired salience [103]. However, most real-life settings are not as constant as laboratory conditions—for good reasons—aim to establish. The conspicuity of the cues an organism encounters in nature varies across episodes [104], implying that ϕ variation may influence the ε trajectory [105]. This can be derived from formalizations of dual salience but has been poorly explored. To study the interaction of these factors, one can examine how the order in which an organism faces increasingly easy or hard discriminations shapes its performance. Easy and hard, when ascribed to a discrimination, can be operationalized through the perceptual distance between two cues, which instantiates how conspicuous their difference is, and thus the unacquired salience (ϕ) of the cue set.

In a seminal series of studies Treviño *et al.* [106] have taken a bold step towards understanding this phenomenon, with relevant implications. They trained mice (*Mus musculus*) to navigate a ‘Y’ maze to escape from a water tank. The escape platform was hidden but signalled by a specific cue (C+), while the alternative alley, signalled by varying cues (C–), led to a platformless end. To escape effectively, the mice had to orient their visual field towards the cues in order to select the correct alley. The authors manipulated the degree of similarity between the C+ and C– cues, which can be interpreted as a measure of unacquired salience. When C+ and C– were highly similar, the path to the platform was scarcely salient, and when they were very different, the path was highly salient. The critical findings emerged from comparing two conditions. In one, the similarity between the escape and no-escape cues increased across trials; in the other, it decreased. The former can be conceptualized as an easy-to-hard condition and the latter as a hard-to-easy condition.

In Treviño *et al.'s* [106] experiments, two groups of mice received identical sets of C+/C– pairs across training, differing only in the order of presentation. One group started with easy (low similarity) discriminations and progressed to hard (high similarity) ones and the other progressed in the opposite direction. Group performance was indexed by the proportion of correct choices across the trial sequence. Training with decreasing similarity led to higher maximum performance than training with increasing similarity. Training with increasing similarity, however, produced less biased overall performance and successful discriminations (above chance) even when the cues were highly similar, whereas the hard-to-easy group performed at chance level when facing the equivalent similar cues. Because the cue deck (the whole collection of C+/C– pairs) is matched and only the ordering changes, the overall difference in performance between groups cannot be attributed to discriminability *per se* and must instead reflect how the salience trajectory unfolded over training.

Another finding from the same Treviño studies was that mice trained under decreasing-similarity conditions were slower to stop responding when the platform was removed, taking longer to abandon their search for the absent platform. Salience would be expected to rise in the increasing-similarity group because its later episodes were partially reinforced, the error rate having increased over the final segment of training for this group. Esber & Haselgrove [39] hold that partially reinforced cues are more readily learned about, which would account for the faster extinction seen here. The decreasing-similarity group, by contrast, was encountering long streaks of reinforced episodes. Salience-loss-from-predictability models predict that such streaks lower salience, and the Esber–Haselgrove model agrees for this regime, since once a cue becomes a reliable predictor the aggregate prediction error shrinks, and salience falls with it. On this account, the increasing-similarity group ends training with higher salience than the decreasing-similarity group, and the higher salience supports faster learning of the extinction contingency. The high salience of the cue could therefore have magnified the negative prediction errors when it failed to predict the platform, potentially driving rapid task disengagement (see [47]). Esber & Haselgrove [39] note that the partial-reinforcement rise in salience depends on the amount of training, since over long regimes salience can instead be lost on unreinforced episodes, so the effect is not guaranteed in every preparation.

The partial-reinforcement extinction effect (PREE) is often invoked to explain resistance to extinction, which makes PREE an obvious alternative to consider here. The two accounts, acquired salience and PREE, do not coincide regarding predictions. The Esber–Haselgrove model predicts that reinforced–non-reinforced alternation raises salience and so speeds learning of the extinction contingency, whereas PREE predicts that the same alternation increases the persistence of conditioned value. The accounts also rest on different conditions. Classic PREE involves a constant cue that is alternately reinforced and not, while Treviño *et al.*'s [106] preparation varied the cue deck incrementally across trials, so the conditions do not clearly map onto one another. A second mismatch lies in the measurements. The canonical conditioned response in PREE work is a stimulus-response measure, the probability or vigour of responding, whereas the index here is persistence of an orienting and search response, so it is not clear that what extinguishes in this task is conditioned value in the sense PREE concerns. Setting cue constancy aside and attending only to the reinforced–non-reinforced sequence, the results favour an uncertainty-driven salience account and are at odds with a PREE explanation.

A subsequent reanalysis of the data by Treviño [41], implementing a formal unacquired and acquired salience partitioning approach [39], expanded on the intuitions of Treviño *et al.* [106]. Treviño [41] aimed to determine whether and how the data obtained by Treviño *et al.* [106] could be explained by dynamic salience models of associative learning. Several adaptations of the model were crucial in fitting the data owing to the peculiarities of the behavioural task. First, coding the unacquired component of salience required the configuration of the C+C– set, rather than the detectability of any single cue; specifically, the unacquired salience was coded as a complement of the similarity between C+ and C– (i.e. 1 – SIM^C+,C–^). Second, associative strength was measured by the proportion of mice choosing the correct alley in the Y maze, rather than by average response frequency or vigour indices typically used. Third, Treviño [41] incorporated a salience threshold into the model to modulate the asymptote of learning curves. This modification reflects the assumption that even well-trained learners will perform poorly if effective salience falls below the minimum required for reliable discrimination. The main result of this reanalysis was that a model incorporating both dynamic and static components of salience provided a better fit for the learning curves compared to simpler models. Importantly, this result held even after accounting for model complexity penalties using the Akaike information criterion [107].

Treviño [108] adapted a visual search task for humans to further explore learning curve trajectories under easy-to-hard and hard-to-easy conditions. In this task, participants were required to detect the presence or absence of a relevant cue among distractor cues that varied in similarity to the target cue. The learning curves observed in this study markedly resembled those reported by Treviño *et al.* [106] in mice. Moreover, these learning curves were best predicted by the salience formulation proposed by Esber & Haselgrove [39], which incorporates both unacquired (i.e. static) and acquired (i.e. dynamic) components. Treviño [108] also analysed the response times of the participants using a diffusion decision model to examine how they managed the speed–accuracy trade-off in the task. This analysis revealed that the easy-to-hard condition facilitated faster accumulation of perceptual information. Such a result aligns with the findings of Treviño *et al.* [106], which showed that while mice trained under hard-to-easy conditions achieved higher maximum performance in terms of sheer discrimination, they were less efficient overall in the perceptual learning task.

Overall, the results of the series of studies by Treviño *et al.* [106] support the idea that cue associability evolves as agents interact with cue–outcome couplings, and this phenomenon manifests itself across mammalian taxa (here, rodents and primates). The superiority of Esber & Haselgrove's [39] salience-partitioning approach in fitting learning curves demonstrates that sheer discriminability and subjective salience are dissociable but interdependent. Most importantly, training regimes composed of identical cue decks but in different sequential orders can lead to distinctive learning trajectories, ultimately yielding different perceptual accuracy. This provides a principled basis for understanding classic studies of *errorless discrimination*, using protocols that fade in or out ancillary conspicuous cues to achieve fine perceptual capabilities that otherwise would be hard to learn (e.g. [109]). For example, when teaching children the alphabet, it is common to start with a grapheme accompanied by a picture and a particular colour, then present the grapheme with colour alone and finally the bare grapheme. Finally, the findings by Treviño *et al.* [106] also challenge cognitive and neurobiological models that posit static stimulus representations to explain perceptual performance.

## 7. Neural substrates of salience dynamics

Because behaviour is constrained by neural architecture, its flexibility ultimately reflects the nervous system's capacity to reorganize (i.e. plasticity). By extension, behavioural meta-malleability requires neuronal meta-plasticity. The scheme posited in §2 can be transposed onto the neural level. Neural plasticity relies on mechanisms that regulate synaptic efficacy (level 0), such as long-term potentiation and long-term depression (level 1). The term *meta-plasticity* (level 2) refers to processes with their own dynamics that layer over basic plasticity, tuning the range within which synaptic efficacy can change [110]. If salience modulates learning by gating or regulating behavioural change, then identifying the neural mechanisms underlying salience dynamics could enhance our understanding of neural meta-plasticity. The sections below survey recent and established findings concerning neural substrates of salience modulation, including attenuation, gain, prediction-error gating and dynamic updating.

### 7.1. Salience dynamics and the cortex

In vertebrates, the integration of sensory information from different modalities to produce actions congruent with the environment (e.g. [111]) occurs in the forebrain. In mammals, layered structures like the primary cortices receive sensory input relayed by thalamic nuclei and transmit this information to higher-level cortical areas (e.g. [112]). However, increasing evidence has shown that activation of primary cortical areas is far from a static representation of environmental cues [113,114].

If salience is to be dynamic, some higher-order structure must be able to reach into and influence the nodes where perceptual activity occurs; otherwise, experience would have no route by which to reshape what is perceived. Rather than being solely driven by thalamic input, primary cortical activity is continuously modulated by feedback from higher cortical areas in a top-down manner [115]. Because this requirement is architectural rather than a property of any particular brain, it should hold even where the structures we usually credit it to are absent. This higher-order regulation has been observed in primary sensory regions across species, even in those without a cerebral cortex. Accordingly, activity in the optic lobes located in the posterior portion of crab (*Neohelice granulata*) eyes is regulated by higher-order structures [116]. Top-down regulation of primary sensory brain modules is believed to be experience-dependent, shaping how animals perceive the world and enact adaptive behaviours [117]. Perhaps one of the primary functions of this top-down regulation is to reduce long-term responsiveness to familiar cues through a compensatory increase in activity within higher-order cortical regions (e.g. [118])—a hallmark of salience attenuation via habituation-like mechanisms. From an evolutionary perspective, the adaptive value of this mechanism may lie in modulating the threshold at which experiences influence future behaviours, given that memory formation is energetically costly [119].

In the cortex, bottom-up signals are conveyed to higher-order cortical regions, producing gamma-band oscillations when a stimulus is attended to (primarily when it is unexpected; [120,121]). This gamma activity decreases as the stimulus becomes increasingly expected [122]. Note that gamma-band activity arises in response to both unexpected cues and the omission of expected cues [123], that is, upon prediction errors. The adaptive role of this signal is believed to support the reorganization of the neural assembly required for learning. The reduction of gamma-band activity when a cue is no longer unexpected is thought to rely on efference copies that anticipate external inputs propagating from higher-order regions. These descending signals are known to produce slower oscillations—such as beta [124,125] or alpha [126]—rather than gamma, which may attenuate further signal transmission upstream. Therefore, gamma-band cortical activity may serve as a proxy for cue salience, dynamically modified by experience as unexpected cues become expected.

Recent 7T fMRI studies have revealed finer details of cortical signal processing in the human visual cortex. Thomas *et al.* [127] tested whether feed-forward and feedback signals are transmitted at different levels of the cortical laminae. In their study, the primary visual cortex (V1) recordings were divided into three layers: superficial, middle and deep. Upon presenting expected (0.75 probability) and unexpected (0.25 probability) cues, the authors found that expected cues elicited comparable activity across layers, whereas unexpected cues primarily activated the superficial layer. These results support the idea that higher-order cortical cells attenuate upstream signals from predicted cues via synapses in the superficial layers of lower-order regions. This top-down attenuation of predicted cues is a candidate physiological realization of the predictability-driven salience loss formalized in equation (4.6), where the term $k\times \sum V^{\text{pre}\to X}$ subtracts from a cue's salience to the degree that preceding features already predict it. According to the authors, their research contributes to an increasingly detailed understanding of cortical salience modulation, with potential implications for perceptual alterations in psychosis [127].

Beyond modulation through habituation-like attenuation, evidence also suggests that cerebral cortex activity is tuned by experiences involving significance gains (e.g. pairing an affectively neutral cue with a reward) as early as in the primary sensory cortices. For example, Henschke *et al.* [128] found that pairing a visual cue with a reward enhances activity in specific patches of the primary visual cortex (V1) in mice. In their study, two equally reward-paired stimuli elicited similar overall increases in activity but became increasingly differentiated at the level of individual neurons. This cortical enhancement is the neural counterpart of the acquired salience gain (see §4.1) inferred behaviourally when orienting responses to cues of different modalities increased once those cues were paired with food. In another study, Pakan *et al.* [129] showed that, in the absence of visual cues anticipating reward, the neuronal and behavioural responses of V1 were driven by self-motion feedback. These findings further challenge the notion of the cortex as a passive recipient of physical environmental qualities. Instead, the cortex appears to be dynamically tuned to respond differentially to environmental features that are behaviourally relevant, shaping how we orient our percepts and engage with the world.

One important region supporting sophisticated behaviour in mammals is the prefrontal cortex, for which rodent associative-learning research has focused on the medial portion. The medial prefrontal cortex (mPFC) has been studied mainly through two functionally distinct modules, the prelimbic (PL) and infralimbic (IL) cortices, though other regions have also been examined [130]. The PL exerts top-down modulation of lower-order behavioural-execution circuits by selecting higher-order cues that contextualize cue–outcome relationships [131]. The functional organization of the mPFC across the dorsoventral axis (PL, more dorsal; IL, more ventral) is contested. One approach holds that the PL engages with the environment in more abstract ways (more context-dependent, less tied to current cues), while the IL is more involved in automatized, habitual behaviour [131]. A different view casts both regions as context-dependent down-regulators of associative states, with the PL modulating appetitive states and the IL aversive ones (for a synthesis, see [56,132]). What seems uncontroversial is that the mPFC houses a node in a higher-order behavioural-regulation circuit that operates by modulating thresholds and attending to informative contextual cues.

### 7.2. Salience dynamics and subcortical forebrain structures

Sensory information is transmitted to the cortex primarily via the thalamus. However, what reaches the cortex is not a fixed imprint of sensory input; rather, the thalamus selectively amplifies unpredicted sensory information. This occurs by enhancing the salience of cues that violate an agent's expectations—known as prediction error signals. Furutachi *et al.* [133] recently demonstrated that in the visual case, this process is mediated by a cooperative interaction between pulvinar cells of the thalamus and inhibitory interneurons in the cortex. Notably, the co-activation of these cell populations preferentially promotes the activity of V1 neurons that are most selective for the features of an unexpected stimulus. This mechanism probably acts as a gating system, amplifying feed-forward sensory input in proportion to how much a stimulus deviates from the prior context-specific experience. As a result, relevant information is transmitted more effectively, supporting neural reorganization that enables adaptive behaviour in similar contexts.

There are other sources of evidence that the thalamus actively modulates the transmission of perceptual signals to the cortex, rather than serving as a passive relay of sensory input [134,135]. For example, a study by Mo *et al.* [136] demonstrated that transthalamic (cortico-thalamo-cortical) circuits are essential for propagating haptic signals from the primary to the secondary somatosensory cortex. Specifically, they found that optogenetic disruption of this circuit impaired performance in a texture-discrimination task in mice. Importantly, top-down (cortico-thalamic) pathways were found to be critical for texture selectivity in neurons of the secondary somatosensory cortex, a selectivity established over several trials of learning texture-outcome contingencies. This finding challenges the corticocentric conceptualization of perception mapping.

The thalamus not only modulates behavioural updating through its canonical connections with the neocortex. A study by Zhu *et al.* [137] in mice demonstrated that activity in the paraventricular thalamus—a region not directly connected to the sensory cortices—decreased with repeated presentations of odour cues in a cue-specific and long-lasting manner. Moreover, silencing the paraventricular thalamus during anticipatory cues in a go/no-go task led to reduced responding during the go cue and increased responding during the no-go cue relative to controls. The authors concluded that these findings support the idea that the paraventricular thalamus conveys salience information, gating associative learning driven by both positive and negative prediction errors. Although the paraventricular thalamus lacks direct connections to the sensory cortices, it is strongly connected to key plasticity centres such as the amygdaloid complex and the nucleus accumbens, which may explain its role in learning. Furthermore, this structure receives dense innervation from the hypothalamus and brainstem, suggesting that its function is modulated by contextual arousal and homeostatic states [137].

### 7.3. Salience dynamics and neuromodulation systems

Bidirectional communication between the thalamic nuclei and the cortex does not function in isolation [138,139]. Evidence suggests, for instance, that the lateral geniculate nucleus—the primary visual thalamic nucleus—operates in concert with neuromodulators supplied by brainstem nuclei. Importantly, the interaction between these brain regions is nonlinear. Noradrenaline (NE) inputs from the locus coeruleus to the thalamus simultaneously promote pupil dilation, presumably enhancing visual acuity across different time scales [140]. Specifically, while pupil dilation facilitates retinogeniculate communication, NE-induced tonic spikes enhance thalamocortical communication.

Intriguingly, visual acuity is modulated not only through thalamic control of pupil diameter but also by hypothalamic input to the retina. Recent findings indicate that the hypothalamus sends histaminergic signals to the retina to enhance light sensitivity. Warwick *et al.* [141] presented converging evidence supporting this, including findings that histaminergic input alters retinal ganglion cell activity and improves the detection of fast-moving images in mice, and that histaminergic antagonism of hypothalamic cells reduces optomotor responses in mice. Furthermore, systemic antihistamine administration modulates human visual field sensitivity. Collectively, these findings reveal that visual processing, even at the retinal level, is closely modulated by both overt (pupil diameter) and covert (histaminergic input) top-down signals. That such modulation reaches the retina is notable because the structural requirement that higher-order systems access perceptual nodes is shown to extend beyond the cortex to the most peripheral locus there is: the sensory surface itself.

Monoaminergic neurons in the brainstem modulate both long-term neural reorganization and real-time adaptation. Among them, dopamine has been considered essential for associative learning [142]. Phasic dopamine activity surges when sensory receptors detect reward-related cues.^8^ This activity contributes to behavioural updating by inducing long-term synaptic plasticity through signed prediction errors, which are positive when a reward is presented and negative when an expected reward is omitted. Beyond encoding reward prediction errors, phasic dopamine responses also arise for novel or otherwise salient cues that are affectively neutral, and they accompany spontaneous behavioural fluctuations without explicit reward [144], suggesting a broader role in salience processing.

A series of experiments by Kutlu *et al.* [145] demonstrated that both novelty and physical intensity influence dopamine release in the nucleus accumbens core, supporting the idea that dopamine activity is related to perceived salience. Moreover, Kutlu *et al.* [145] selectively stimulated the nucleus accumbens and observed a reduction in defensive responses typically elicited by a fear-conditioned cue. This latter finding was interpreted as evidence for salience encoding by dopaminergic signalling, as the observed effect resembles external inhibition—the attenuation of conditioned behaviour in response to a cue owing to the introduction of a salient companion cue (cf. [62]).

A subsequent study by Kutlu *et al.* [146] provided more conclusive evidence supporting the causal role of accumbal dopamine release in salience updating. In this study, optogenetic stimulation of the dopamine terminals in the nucleus accumbens core during a latent inhibition protocol (i.e. repeated presentation of the cue without any affectively loaded stimulus) enhanced acquisition retardation in a subsequent stage without optogenetic stimulation, while leaving the response to a previously established excitatory cue unaltered (i.e. ruling out both external inhibition and conditioned inhibition). This finding builds on Kutlu *et al.* [145], suggesting that dopamine activity in response to cue presentation enhances perceived salience, which in turn diminishes the subsequent perceived salience of that specific cue. In addition, an emerging body of evidence suggests that, beyond encoding affective values, prediction errors or perceived salience (e.g. novelty, physical intensity), dopamine also plays a role in encoding cue identity. This conclusion is supported by findings showing that dopamine activity in response to prediction errors occurs not only for reward but also for specific features of affectively neutral sensory stimuli (see [147], for a recent review).

Norepinephrine (NE) and acetylcholine (ACh) probably play a more prominent role in the encoding of salience, particularly in attention and sensory gating. To establish the neural basis of salience within associative learning frameworks, specific criteria must be met. Salience encoding would be demonstrated by evidence of unsigned prediction errors—neural activity whose absolute magnitude scales with expectancy violations, regardless of valence [148]. In addition, confirming salience encoding requires ruling out affective specificity and motivational confounds. Finally, in alignment with learning models, it is crucial to show that salience acts as a gateway to behavioural updating.

ACh participates in associability dynamics within a distributed circuit. The central amygdala, the striatal-like portion of the amygdaloid complex, is critical for registering the surprise that drives salience increases [149]. Signals to enhance the associability of cues (driving faster learning) are conveyed by cholinergic projections from the substantia innominata of the basal forebrain to the posterior parietal cortex (PPC), and reorganization of the PPC is what allows retention of these associability changes [149]. Holland & Schiffino [149] argue that this mechanism for salience increase is dissociable from the salience decrease seen in latent inhibition, which parallels the separate update paths for salience gains and pre-exposure-driven losses in the Esber–Haselgrove model.

It is widely known that dopamine neurons are modulated by food deprivation, as shown by the findings of Konanur *et al.* [150] that deprivation increases reward-cue-induced phasic activity in dopamine neurons. The phasic dopamine activity that fosters the reorganization necessary for behavioural change is also modulated by acquired salience, which is encoded as dips in ACh activity in the interneurons of the nucleus accumbens. A recent study by Zhang *et al.* [151] showed that differential cholinergic activity associated with cues signalling either reward or its omission emerged downstream of cue-dependent dopamine activity. This finding was interpreted as salience acquisition occurring only after the cues gained biological relevance. Furthermore, once cholinergic dips were established upon cue presentation, their magnitude in one trial predicted the magnitude of dopamine signalling in the subsequent trial. This observation supports the role of ACh in modulating dopamine updates that, in turn, facilitate the performance of learned behaviours. In addition, when subjects were exposed to different discrimination scenarios, cholinergic dips occurred progressively earlier with each consecutive set of cues, suggesting their contribution to enhancing attention to task-relevant features with repeated experience. Finally, the authors ruled out that cholinergic dips were primarily involved in either motivation or reward anticipation, establishing their role as a key factor in salience endowment.

Regarding NE, a study by Lui *et al.* [152] demonstrated that optogenetic stimulation of locus coeruleus neurons during the extinction of appetitive conditioned behaviour did not affect learning rates but enhanced the expression of extinction in a spontaneous recovery test. At that point, extinction may have been facilitated either by an increase in salience or by the promotion of an aversive state (akin to punishment). Crucially, the latter explanation was ruled out by showing that photostimulation of locus coeruleus neurons did not induce place aversion in a conditioned place preference paradigm. Lui *et al.* [152] also reported that photoinhibition of these neurons did not produce the opposite pattern (i.e. impaired expression of extinction), raising questions about the causal role of locus coeruleus NE neurons in extinction. In addition, the fact that acquisition rates were unaffected casts doubt on whether NE enhances salience specifically. An alternative is that NE strengthened consolidation of extinction rather than any factor governing the updating mechanism engaged during the task, which would equally fit the reduced spontaneous recovery.

A follow-up study by the same group provided further insight. Brink *et al.* [153] administered clonidine, an NE autoreceptor agonist, directly to the locus coeruleus to inhibit downstream NE signalling. Subjects exposed to clonidine during extinction trials exhibited a faster reacquisition rate compared to saline controls, suggesting relatively impaired extinction learning. Moreover, subjects who underwent overexpectation training under the influence of clonidine failed to show the typical reduction in responding to the target stimuli, unlike those administered saline. This finding extended the previous results, indicating that the impairment was not specific to extinction but reflected a broader deficit in learning from negative prediction errors. Nevertheless, the common feature of these paradigms can also point to a confound that invites an alternative explanation. Both extinction and overexpectation learning are known to rely on negative prediction errors, so it is no less plausible that what NE signalling really does is enhance those negative prediction errors. Prediction errors, however, are linked to the affective states of outcomes. A negative prediction error for an appetitive outcome is aversive, and one for an aversive outcome is appetitive [46]. In these paradigms, the relevant negative prediction error and the aversive state it constitutes are not separable, so Lui *et al.*'s [152] manoeuver for ruling out an aversive-state account of extinction enhancement rules out the negative-prediction-error account by the same token. Taken together, though a consolidation account of Lui *et al.* [152] remains unexcluded, the findings of Lui *et al.* [152] and Brink *et al.* [153] point to a role for NE signalling in salience-related mechanisms that regulate the neural reorganization underlying the suppression of conditioned responding.

## 8. Concluding remarks and future directions

Behaviour is malleable, and salience serves as a gate that controls the reorganization mechanisms that drive behavioural change. Because salience is itself dynamic, behaviour can be regarded as meta-malleable. In this paper, we review evidence that positions salience as an enabling factor for learning and highlights dynamic salience as a robust phenomenon observed across diverse laboratory paradigms. The neural underpinnings of salience are increasingly being delineated, and future studies using advanced imaging and manipulation techniques will further clarify how the gating of reorganization mechanisms is implemented in biological systems.

At this point, a more concise framing of the implications of dynamic salience and meta-malleability is warranted. First, basic behavioural malleability implies that two similarly constituted individuals may behave differently at a given time because their past experiences diverge. In this case, learning is the mechanism that allows ontogenetic paths to fork when individuals encounter different circumstances. Meta-malleability goes further. It requires recognizing that the mode of engagement with a circumstance (i.e. how an individual perceives, attends to or interacts with it) is itself influenced by prior experience. Under this view, even when initial states and current circumstances are identical, individuals may still diverge because their history has altered how they engage with the environment. Thus, learning experiences that appear similar from the outside do not affect individuals equally; they only seem equivalent at the surface level, but differ in their perceptual subjectivity. This does not depend on any ineffable property of organisms but on the active—sometimes covert—ways in which they interact with their surroundings.

A key implication of dynamic salience is that it can amplify small initial differences into distinct behavioural trajectories over ontogeny, thus contributing to behavioural individuation. Even when individuals start under apparently equivalent conditions, differences in how strongly they weight cue–outcome experiences can make their developmental pathways diverge. Heightened salience would make particular experiences disproportionately influential, bending behavioural trajectories to enter a state transition, thus providing a mechanism for faster behavioural drift. Lower salience, by contrast, would create higher behavioural inertia, which implies that state transitions require greater amounts of experience, fostering earlier stabilization around particular profiles. Importantly, associative learning is not limited to reflexive responses such as salivation; it also shapes the preferences and aversions that guide individuals towards particular niches, which in turn reshape them [154]. These adjustments alter tendencies to approach or avoid and, therefore, the situations in which individuals place themselves. Over time, feedback between salience, experience and self-selected circumstances can amplify subtle variation into pronounced individuality (see [155–158]). In this way, dynamic salience offers a plausible and formally tractable mechanism that complements current work on the development of animal personality and behavioural individuation [159–161]. More broadly, making explicit the mechanisms that underlie behavioural meta-malleability speaks directly to long-standing nature–nurture debates (e.g. [132]), by showing how similar biological endowments and environments can still yield divergent behavioural paths.

An important next step is to extend the bridge between abstract equations in dynamic salience models and the neural machinery that could implement them. To do so, dynamic salience should be embedded in structured models rather than treated as a free-standing term in a formula. This implies assigning its components to identifiable nodes and signals in neural circuits: where prediction errors are generated, where salience signals arise and how they modulate plasticity or gain in downstream pathways. Several such mappings are already in hand. For example, the predictability-driven salience-loss term of equation (4.6) corresponds to the cortical attenuation of predicted cues reviewed in §7.1, where higher-order areas suppress upstream signals from cues the context already predicts.

In addition, if we are committed to moving beyond the stationary computer metaphor (see [162]), these nodes and signals should be embedded in agent models that actively regulate their perceptual activity by navigating their environment (e.g. [75,163,164]). One natural starting point is to formalize these ideas as hierarchical control systems with higher levels that regulate the salience of lower-level inputs. However, accumulating evidence for non-transitive organization suggests that the architecture supporting behaviour may be better understood as heterarchical rather than as a simple hierarchical pyramid [165]. For example, while salience certainly modulates learning, acquired associative value also feeds back into salience (see equation (4.2)). Similarly, drive does not modulate salience alone; it also acts on learning directly through outcome-related associability (β). The levels therefore do not form a strict chain in which each speaks only to the one immediately below. Building explicit circuit-level architectures, even in simplified toy systems, would sharpen hypotheses about the timing, localization and neuromodulatory substrates of dynamic salience and generate concrete questions for empirical work.

Dynamic salience may also be relevant for the design of artificial agents. Many applications in machine vision and decision systems require mechanisms that decide which inputs are informative, when internal models should be updated rapidly and when stability should be preserved [166,167]. In this context, dynamic salience could be used to boost action recognition; for instance, to tune when a system tracks a face, a movement pattern or a configuration of cues in a video stream as worth tracking more closely [168], and when new evidence should drive larger parameter updates instead of merely accumulating more of the same [169]. While one goal could be to understand dynamic salience in organic agents, this line of inquiry could also borrow the control principle involved: the guidance of learning towards relevant features [170] by gating plasticity, exploration and computational effort. Exploring such implementations in artificial systems might, in turn, feed back into biological research by revealing which forms of dynamic salience are necessary for robust performance.

## Data Availability

The scripts required to reproduce the simulation-based figures included in this article are provided in the supplementary material associated with this submission. Data and relevant code for this research work are stored in GitHub: http://github.com/RSosa-BehavioralScience/dynamic_salience and have been archived within the Zenodo repository [171].

## Footnotes

^1^
While this taxonomy of salience may resemble other prominent frameworks of attention, such as the pre-attentive salience versus voluntary attention model by Itti & Koch [1], it explicitly links to the origin (learned versus unlearned) of attentional capture rather than the implementation of salience maps from different sources of information (bottom-up versus top-down). In the latter, the intrinsic conspicuity of cues interacts with task demands, with different neural pathways proposed to maximize information gain for each.

^2^
It is worth remembering, as George Box [15] famously noted, that all models are wrong, but some are useful. Associative models, like all scientific models, are merely simplified approximations of the phenomena they aim to represent. Although these theoretical constructs may not always perfectly align with observed reality, they have proven to be quite effective in both describing behavioural change and generating insights about the potential mechanisms intervening in this process (e.g. prediction error; [16,17]). This effectiveness should not obscure the need to assess where the models hold and where they fail (see [18]).

^3^
Segmenting units of analysis into trials has been a strategy that most classical associative learning theorists have adhered to in order to emphasize the importance of pairings between the cue and the significant outcome [21]. However, alternative approaches (e.g. temporal difference models; [13,22,23]) account for the continuous nature of time and include the intervals between cue–outcome pairings in the computation of associative learning processes.

^4^
In addition, attention has been characterized in so many ways (e.g. as a filter, a limited source, a spotlight and an integrator; [25]) correspondingly implying so many ways to measure it (e.g. selecting cues, ignoring cues, persevering, multi-tasking; [26]). In this paper, we describe two forms of operationalized behavioural tendencies related to attention, infotaxis and associability (see the main text in this section and the one below), which are tractably related to salience.

^5^
This notation is used for describing the minimum elements of trials in conditioning protocols. Specifically, a plus sign next to letters that identify cues indicates that an outcome follows each cue, whereas a slash represents the independent presentation of different types of trials.

^6^
While we are adhering to the terms coined by Esber & Haselgrove [39], other authors have used different nomenclatures for the same constructs. For example, Pearce & Hall [40] referred to them as ‘salience’ and ‘associability’, respectively, and Treviño [41] opted for ‘effective’ and ‘acquired’ salience to describe these concepts.

^7^
This assumes that invigourating the ‘teaching signal’ to engender behavioural reorganization—i.e. what salience does—represents a response from the organism itself. However, unlike other activities of the organism, these responses are usually hidden (covert) and thus require special tests to ascertain them through behavioural post-trial performance upon presenting the target cue. Therein lies the importance of examining unacquired or acquired differences in associability.

^8^
The canonical function of dopamine is contested on the grounds that performance confounds have not been appropriately ruled out as an alternative to a prediction-error role. Phasic dopamine may instead track the force exertion that reward retrieval requires, so that activity attributed to a teaching signal reflects ongoing movement [143].
